# Finding the priority and cluster of inflammatory biomarkers for infectious preterm birth: a systematic review

**DOI:** 10.1186/s12950-023-00351-0

**Published:** 2023-07-24

**Authors:** Jiayi Li, Jianrong Ge, Na Ran, Changwu Zheng, Yumeng Fang, Danna Fang, Qian Yang, Yeling Ma

**Affiliations:** 1 School of Medicine, Shaoxing University, Shaoxing, Zhejiang 312000 China; 2 NHC Key Lab of Reproduction Regulation, Shanghai Engineering Research Center of Reproductive Health Drug and Devices, Shanghai Institute for Biomedical and Pharmaceutical Technologies, Shanghai, China

**Keywords:** Preterm birth, Inflammatory factor, Biomarker, Prioritization, Maternal blood

## Abstract

Infectious preterm birth (PTB) is one of the most important causes of perinatal death. It is difficult to find reliable biomarkers accurate to gestational weeks for infectious PTB prediction clinically. Infectious PTB is found usually accompanied with immune imbalance. Thus, the systematic study to find the priority of inflammatory biomarkers and innovative inflammatory clusters for infectious PTB prediction is urgently needed.

This systematic study that focused on the inflammatory clusters and infectious PTB in the PubMed database was analyzed by using the criteria of the Population, Intervention, Comparison, Outcome, and Study design (PICOS) framework according to the recommendations of preferred reporting items for systematic reviews and meta-analysis (PRISMA).

The network meta-analyzed results showed that the prioritization of the inflammatory factors for infectious PTB prediction is soluble tumor necrosis factor receptor 2 (sTNFR2) > tumor necrosis factor α (TNFα) > interleukin-10 (IL-10) > interleukin-6 (IL-6) > C-reactive protein (CRP) > interleukin-1β (IL-1β). Furthermore, the results also indicated that global consideration of multiple inflammatory factors, such as CRP/IL-1β/IL-6 biomarker cluster in gestational 27–34 weeks, and the tumor necrosis factor/nerve growth factor (TNF/NGF) family during gestational 25–33 weeks, were potential biomarker clusters that specific for infectious PTB prediction.

This study systematically pointed out prioritization of the inflammatory factors for infectious PTB prediction. The results also provided evidence that maternal inflammatory clusters can predict infectious PTB occurrence at accurate gestational week. The global consideration of multiple inflammatory factors at accurate gestational age is highlighted.

**Supplementary Information:**

The online version contains supplementary material available at 10.1186/s12950-023-00351-0.

## Background

One of the main causes of perinatal death is preterm birth (PTB), which is defined as a delivery before 37 weeks of gestation [1, 2]. According to the World Health Organization (WHO) research from 2019, 14.8 million newborns were born with PTB at a rate of 10.6% [3]. Infection and infection caused inflammation are the main causes of PTB [4].

Maternal infection can release inflammatory factors into the maternal peripheral circulation [4, 5]. Microorganisms can induction parturition under infectious condition [6], microorganisms and their products can activate the toll-like receptor and induce the production of chemokines, cytokines, prostaglandins, and proteases [7–9]. During intra-amniotic infection, the microorganisms can attack the fetus and cause brain or lung problems by triggering a fetal systemic inflammatory response. Under this circumstance, the infants born immaturely before term [10]. In addition to intra-amniotic infection, extra-uterine infections, such as malaria, can also induce PTB by creating an inflammatory imbalance [4].

The protein biomarkers are most common for infectious PTB. For example, the inflammatory factors that mentioned in this study included sTNFR2, TNFα, IL-10, IL-6, CRP, and IL-1β, they could be induced by infection, and highly expressed in infectious PTB [11–19]. The detection method of these protein markers is enzyme-linked immunosorbent assay. The other biomarkers for infectious PTB are not common. For example, microRNA-142 transcript is one of the microRNAs that associated with occurrence of PTB [20]. The detection method of microRNA biomarkers is transcriptome profiling after sampling. In addition, gene set is also found participated in occurrence of PTB [20], and the detection method is genome profiling after sampling. Based on this, the detection of protein biomarkers is much cheaper and more convenient than other biomarkers.

Inflammatory factors in maternal peripheral circulation can show the pregnant status to a certain extent. Over the last several decades, the expression levels of several inflammatory factors have been found to be significantly different between infectious PTB and normal pregnancy. sTNFR2 and TNFα have been found participated in infection caused inflammation [11, 12] and highly expressed in infectious PTB [13, 21]. Interleukins, such as IL-10, IL-6, and IL-1β are essential cytokines promoting inflammation after infection [16] and dramatically upregulated in infectious PTB patients [14, 15]. CRP significantly increases after infection [18], and always be found upregulation in infectious PTB patients [17]. Whether these inflammatory factors can be potential biomarkers to predict the occurrence of infectious PTB needs extensive analysis. This study systematically summarized the published evidence by meta-analysis to find the reliable inflammatory biomarkers for infectious PTB prediction. We found there were 17 acceptable randomized control trials analyzing the potential biomarkers for infectious PTB. We summarized that there were 19 inflammatory biomarkers inside, which included interleukins, interferons, and cytokines, etc. Among these 19 inflammatory biomarkers, there were 2 inflammatory biomarkers significantly upregulating in PTB maternal blood at the time of delivery and 17 inflammatory biomarkers significantly upregulating in infectious PTB maternal blood before delivery. In addition, among these 17 inflammatory biomarkers, CRP, IL-6, and IL-1β were the most frequently studied factors. We further found that CRP, IL-6, and IL-1β might be reliable inflammatory biomarkers for infectious PTB prediction by meta-analysis. Furthermore, CRP/IL-1β/IL-6 cluster were potential biomarkers for infectious PTB patients in gestational 27–34 weeks. The TNF/NGF cytokine family has also been found to participate in the occurrence of infectious PTB during gestational 25–33 weeks. This systematic review provided a fundamental basis for timely prediction and intervention to prevent infectious PTB clinically.

## Methods

### Literature search strategy

Before we did the systematic literature search, we searched for the topic of this study in the PROSPERO database to ensure no similar systematic review was available, and we established the new record in PROSPERO (PROSPERO ID: CRD42022345185). Then we conducted a systematic literature search in the PubMed database from inception until July 2022 to identify the relevant studies on the inflammation biomarkers for PTB patients. Inflammation and PTB related key words were the research terms that were used in this study (Supplementary data 1). We did the Medical Subject Headings (MeSH) for further analysis. The study language was restricted to English. Two authors removed duplicates and evaluated the records independently. The different opinions were discussed with a third author to make a decision.

### Inclusion and exclusion criteria

The inclusion of the references all met the criteria of the Population, Intervention, Comparison, Outcome, and Study design (PICOS) framework according to the recommendations of Preferred Reporting Items for Systematic Reviews and Meta-Analyses (PRISMA) [22]. The inclusion criteria were as follows: (1) population: pregnancies with infectious PTB disorder; (2) intervention and comparison: inflammatory factors in maternal blood between normal and infectious PTB pregnancies; (3) outcome: the blood concentration of inflammatory factors in normal and infectious PTB pregnancies before delivery; and (4) study design: randomized controlled trials. In addition, studies without adequate data (n value, mean value, P value, standard deviation value) were excluded.

### Data extraction

The following information was extracted from the 17 included studies: the first author, publication year, country, sampling time, sample size, and delivery gestational weeks, inflammatory factor, functions. In addition, the P values, means, standard deviation of the means, and sample size in each group were also extracted. In some studies, the statistical data was shown in graphs and bar charts rather than table. The GetData Graph Digitizer software was used to extract the data from the graphs or bar charts. Two authors independently extracted the data. The different opinions were discussed with a third author to make a decision.

### Risk-of-bias and quality assessment

Two authors independently assess the quality of the included studies. Random sequence generation (selection bias), allocation concealment (selection bias), blinding of participants and personnel (performance bias), blinding of outcome assessment (detection bias), incomplete outcome data (attrition bias), selective reporting (reporting bias), and other bias were assessed as “low,” “high,” or “unclear” risk. The risk of bias graph was drawn by the software Review Manager 5.4 (The Cochrane Collaboration, UK). The different opinions were discussed with a third author to make a decision.

### Data synthesis and analysis

The concentrations of different inflammatory factors were analyzed between normal and PTB pregnancies. Studies were divided into a normal pregnancy group and a PTB group. The extracted n value, mean value, and standard deviation value of two groups were imported into Stata MP16 software (Stata Corp, College Station, TX, USA) respectively. The command “db metan” can start forest plot analysis. The command “db metafunnel” can start funnel plot analysis. The command “db metaninf” can start sensitivity analysis. *P*-value < 0.05 was considered to be a significant difference. For network meta-analysis, the command “network map” was used for drawing the network, and the command “sucra prob*, lab(sTNFR2 TNFα IL10 IL6 CRP IL1β)” was used for surface under the cumulative ranking curve (SUCRA) value analysis. The size of the nodes and the thickness of the edges are weighted according to the number of studies evaluating each inflammatory factors and direct comparison in network figure. The cumulative probabilities rank the priority of inflammatory factors for PTB prediction. The curve of each inflammatory factors is the cumulative ranking curve lining out the SUCRA value.

## Results

### Study characteristics

**Fig. 1:**
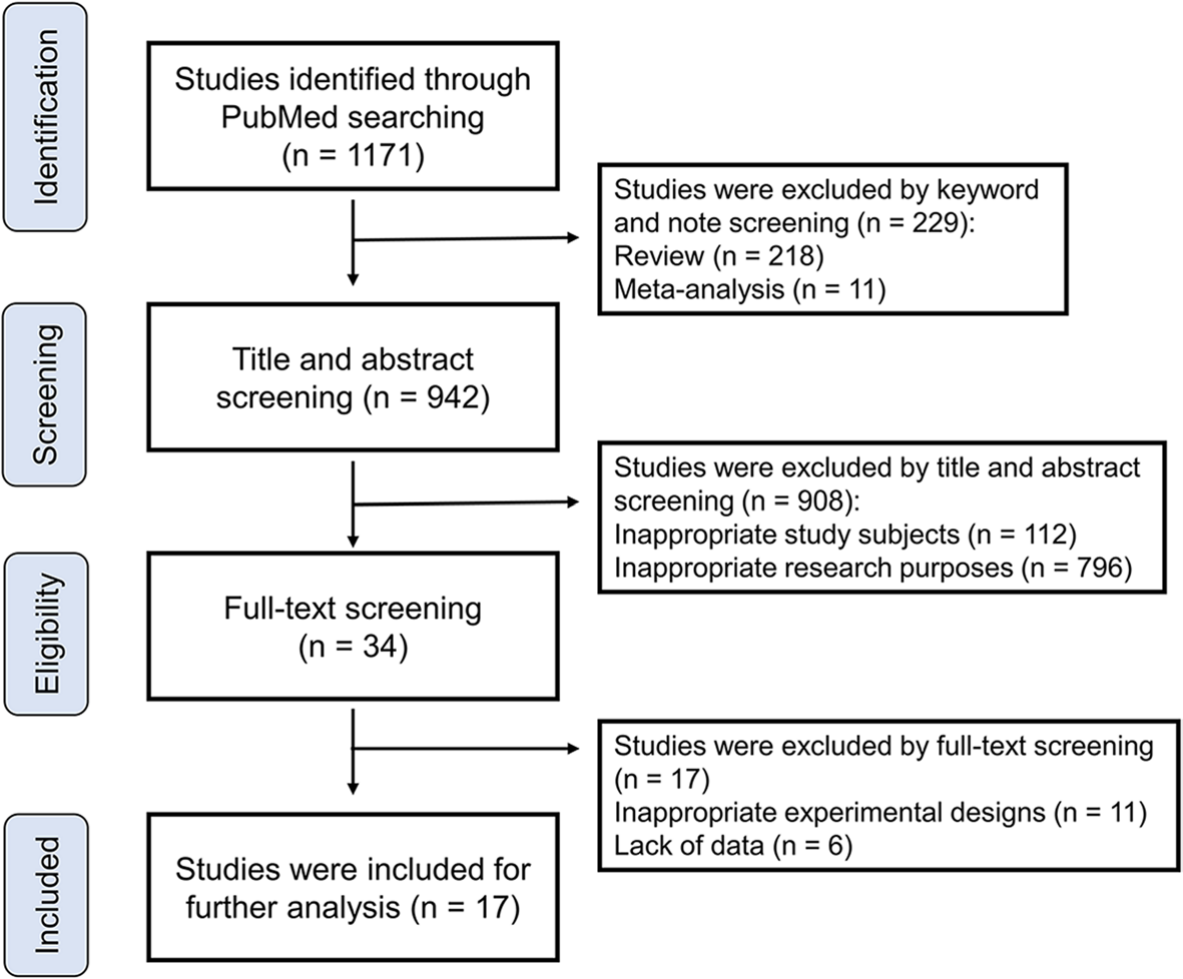
The flow diagram of this study selection process in the meta-analysis

The process of literature search in this study is outlined in Fig. 1. We did our initial search in PubMed databases and identified 1171 records. These records were imported into EndNote for further screening. There were no duplicated records among these records, and the review paper (*n* = 218) and meta-analysis (*n* = 11) were excluded by keyword and note screening. The remaining 942 records underwent further title and abstract screening, and 908 records were removed because of inappropriate study subjects (*n* = 112) and research purposes (*n* = 796). The full text of the remaining 34 studies was carefully reviewed, and 17 studies were excluded because of inappropriate study design (*n* = 11) or lack of adequate data (*n* = 6). Finally, 17 studies that met the inclusion criteria were included in the quantitative synthesis.

Table 1 summarizes the detailed information for the remaining 17 articles. The country, sample origin, gestational weeks for sampling, delivery gestational weeks, significant differences, inflammatory factors, and function of the inflammatory factors, etc. were included.

**Table 1:** The detailed information for the included articles.

Number	Study	Country	Sample	Sampling time	Deliver gestational week	Inflammatory factors	Function	Significance	Sample size (PTB/Term)
1	Ran, Y. (2021).	China	Maternal blood	37 weeks	37 weeks	IL-27p28	Immune modulator	Significantly higher in PTB	15/23
2	Shafiq, M. (2021).	India	Maternal plasma	28-30 weeks	Before 37 weeks	IL-1β	Pro-inflammatory mediator	Significantly higher in PTB	25/193
						IL-17A	Stimulating an inflammatory response	Significantly higher in PTB	
3	Brien, M. E. (2020).	Spain	Maternal plasma	26-29 weeks	Before 37 weeks	CXCL9	Inflammatory activation	Significantly higher in PTB	50/50
4	McDonald, C. R. (2019).	Canada	Maternal plasma	16 weeks	Before 37 weeks	IL-6	Pro-inflammatory mediator	Significantly higher in PTB	55/178
						sTNFR2	Inflammatory activation	Significantly higher in PTB	
5	Escobar-Arregoces, F. (2018).	Colombia	Maternal blood	25-33 weeks	Before 37 weeks	IFN-γ	Immune regulation	No significance	23/23
						TNFα	Inflammatory activation	Significantly higher in PTB	
						IL-10	Pro-inflammatory mediator	Significantly higher in PTB	
						IL-6	Pro-inflammatory mediator	No significance	
						IL-4	Pro-inflammatory mediator	No significance	
						IL-2	Pro-inflammatory mediator	Significantly higher in PTB	
6	Zhu, H. (2018).	China	Maternal plasma	27-34 weeks	Before 37 weeks	IL-6	Pro-inflammatory mediator	Significantly higher in PTB	7/20
						IL-1β	Pro-inflammatory mediator	Significantly higher in PTB	
						CRP	Inflammatory activation	Significantly higher in PTB	
7	Shivakoti, R. (2018).	India	Maternal plasma	21-33 weeks	32-37 weeks	sCD163	Macrophage activation	Significantly higher in PTB	26/81
						I-FABP	Inflammatory activation	Significantly higher in PTB	
8	Wallenstein, M. B. (2016).	USA	Maternal serum	25-33 weeks	Before 37 weeks	sTNFR1	Inflammatory activation	Significantly higher in PTB	34/34
						sTNFR2	Inflammatory activation	Significantly higher in PTB	
						TNFα	Inflammatory activation	Significantly higher in PTB	
						TNFβ	Inflammatory activation	Significantly higher in PTB	
						NGF	Inflammatory activation	Significantly higher in PTB	
9	López, M. (2016).	Spain	Maternal serum	Before 37 weeks	Before 37 weeks	sCD14	Macrophage activation	Significantly higher in PTB	9/27
10	Mesa, F. (2016).	Spain	Maternal plasma	33-37 weeks	33-37 weeks	IL-1β	Pro-inflammatory mediator	Significantly higher in PTB	54/53
						IL-6	Pro-inflammatory mediator	Significantly higher in PTB	
						IL-8	Pro-inflammatory mediator	Significantly higher in PTB	
						IL-10	Pro-inflammatory mediator	Significantly higher in PTB	
11	Perunovic, N. (2016).	Serbia	Maternal serum	Before 37 weeks	Before 37 weeks	IL-1β	Pro-inflammatory mediator	No significance	52/52
						IL-6	Pro-inflammatory mediator	No significance	
						TNFα	Inflammatory activation	No significance	
12	Hastie, C. E. (2011).	UK	Maternal serum	24-37 weeks	24-37 weeks	CRP	Inflammatory activation	Significantly higher in PTB	21/27
13	Sorokin, Y. (2010).	USA	Maternal serum	24-32 weeks	Before 37 weeks	IL-6	Pro-inflammatory mediator	Significantly higher in PTB	22/25
						CRP	Inflammatory activation	Significantly higher in PTB	
14	Torbé, A. (2007).	Poland	Maternal serum	24-36 weeks	31-37 weeks	IL-1β	Pro-inflammatory mediator	Significantly higher in PTB	18/29
						IL-6	Pro-inflammatory mediator	Significantly higher in PTB	
15	Skrablin, S. (2007).	Croatia	Maternal plasma	27-34 weeks	27-34 weeks	IL-6	Pro-inflammatory mediator	Significantly higher in PTB	20/20
						IL-1β	Pro-inflammatory mediator	Significantly higher in PTB	
						CRP	Inflammatory activation	Significantly higher in PTB	
16	Catov, J. M. (2007).	USA	Maternal serum	<21 weeks	34-37 weeks	CRP	Inflammatory activation	Significantly higher in PTB	20/20
17	Goldenberg, R. L. (1996).	USA	Maternal plasma	26 weeks	Before 37 weeks	Ferritin	Induce inflammation	Significantly higher in PTB	51/485

### Study quality

**Fig. 2:**
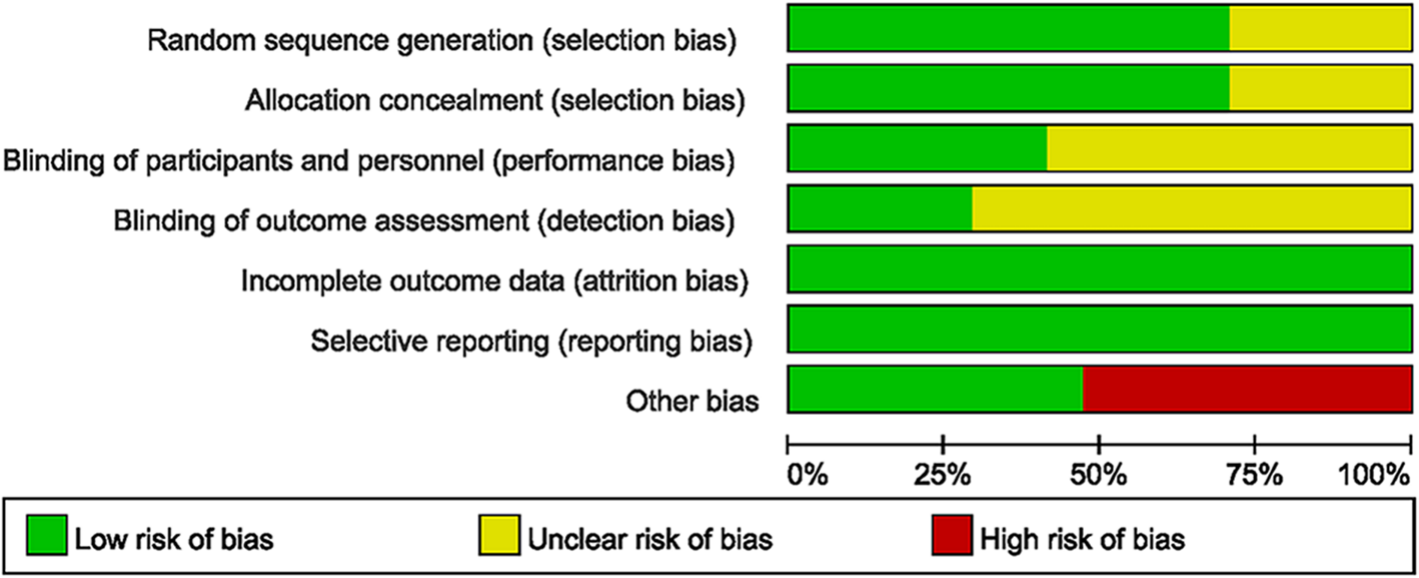
The risk-of-bias assessments of the included studies. The risk-of-bias assessments of the included studies. Random sequence generation (selection bias), allocation concealment (selection bias), blinding of participants and personnel (performance bias), blinding of outcome assessment (detection bias), incomplete outcome data (attrition bias), selective reporting (reporting bias), and other bias were assessed by Review Manager 5.4 as “low,” “high,” or “unclear” risk. Green color indicated low risk of bias, yellow color indicated unclear risk of bias, and red color indicated high risk of bias. About 71% studies have low risk of selection bias, and 29% studies have unclear risk of selection bias. About 41% studies have low risk of performance bias, and 51% studies have unclear risk of performance bias. About 29% studies have low risk of detection bias, and 71% studies have unclear risk of detection bias. All studies have low risk of attrition bias and reporting bias. About 47% studies have low risk of other bias, and 53% studies have high risk of other bias

The qualities of these 17 studies were analyzed in Fig. 2. For the selection bias, 12 studies described the specific methods of randomization. Seven studies on performance bias indicated that double-blind (participants and personnel) was used in these studies. Although the detailed methods of blinding for outcome assessment were not mentioned, the objective measurement of the inflammatory factors is related to double-blinding of participants and personnel. Therefore, the detection of bias was related to performance bias. For attrition bias, 17 studies have complete outcome data. For reporting bias, all the studies have no selective experimental data for high risk. For other bias, 9 studies were judged to have a high risk, including those with a small sample size. Overall, about 71% studies have low risk of selection bias, and 29% studies have unclear risk of selection bias. About 41% studies have low risk of performance bias, and 51% studies have unclear risk of performance bias. About 29% studies have low risk of detection bias, and 71% studies have unclear risk of detection bias. All studies have low risk of attrition bias and reporting bias. About 47% studies have low risk of other bias, and 53% studies have high risk of other bias.

### Main efficacy of meta-analysis

#### The prioritization of the inflammatory factors in infectious PTB prediction is sTNFR2 > TNFα > IL-10 > IL-6 > CRP > IL-1β

**Fig. 3:**
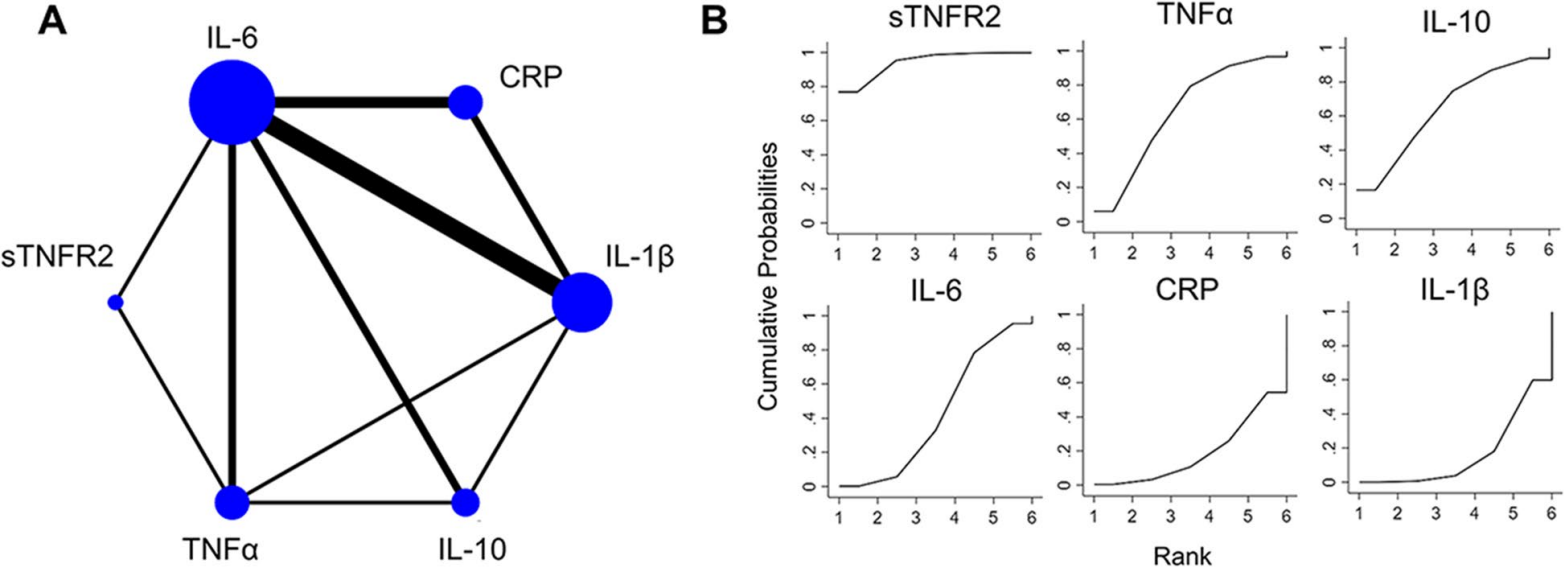
The network and cumulative probabilities of IL-1β, IL-6, IL-10, CRP, TNFα, and sTNFR2. **a** The network of IL-1β, IL-6, IL-10, CRP, TNFα, and sTNFR2. The size of the nodes and the thickness of the edges are weighted according to the number of studies evaluating each inflammatory factors and direct comparison, respectively. **b** The cumulative probabilities of IL-1β, IL-6, IL-10, CRP, TNFα, and sTNFR2 to rank the priority for PTB prediction. The curve of each inflammatory factors is the cumulative ranking curve lining out the SUCRA value. (IL-1β indicated interleukin-1 beta, IL-6 indicated interleukin-6, IL-10 indicated interleukin-10, CRP indicated C-reactive protein, TNFα indicated tumor necrosis factor α, and sTNFR2 indicated soluble tumor necrosis factor receptor 2, SUCRA indicated surface under the cumulative ranking curve)

Among the included 17 studies, 1340 normal and 502 infectious PTB pregnancies were analyzed, and 19 inflammatory factors were studied in the peripheral blood of pregnant women. In the peripheral blood of normal and PTB patients, C-reactive protein (CRP), interleukins (IL-1β, IL-2, IL-4, IL-6, IL-8, IL-10, IL-17A, IL-27p28), tumor necrosis factor (TNF)/nerve growth factor (NGF) cytokine family (TNFα, sTNFR1, sTNFR2, NGF), macrophage related soluble factors (sCD14, sCD163), interferons (IFN-γ), chemokines (CXCL9), ferritin, and intestinal fatty acid binding protein (I-FABP) were analyzed. Among 19 inflammatory factors, 6 inflammatory factors were studied more than twice, and IL-1β, IL-6, IL-10, CRP, TNFα, and sTNFR2 were included. We used network meta-analysis for these 6 inflammatory factors to efficiently rank the priority for infectious PTB prediction. The network and cumulative probabilities of these 6 inflammatory biomarkers were shown in Fig. 3A and Figure 3B respectively. The network analyzed results showed in Table 2 indicated that sTNFR2 had the highest SUCRA value (94%), TNFα had a 64.2% SUCRA value, IL-10 had a 63.9% SUCRA value, IL-6 had a 42.5% SUCRA value, CRP had a 18.9% SUCRA value, and IL-1β had the lowest SUCRA value (16.5%). Thus, the prioritization of the inflammatory factors in infectious PTB prediction is sTNFR2 > TNFα > IL-10 > IL-6 > CRP > IL-1β.

**Table 2:** The SUCRA values of inflammatory factors for PTB prediction.

**Inflammatory factors**	**SUCRA (%)**	**PrBest**	**MeanRank**
sTNFR2	94.0	76.8	1.3
TNFα	64.2	6.0	2.8
IL-10	63.9	16.6	2.8
IL-6	42.5	0.1	3.9
CRP	18.9	0.4	5.1
IL-1β	16.5	0.0	5.2

#### CRP/IL-1β/IL-6 is reliable cluster for predicting the occurrence of infectious PTB in gestational 27–34 weeks

**Fig. 4:**
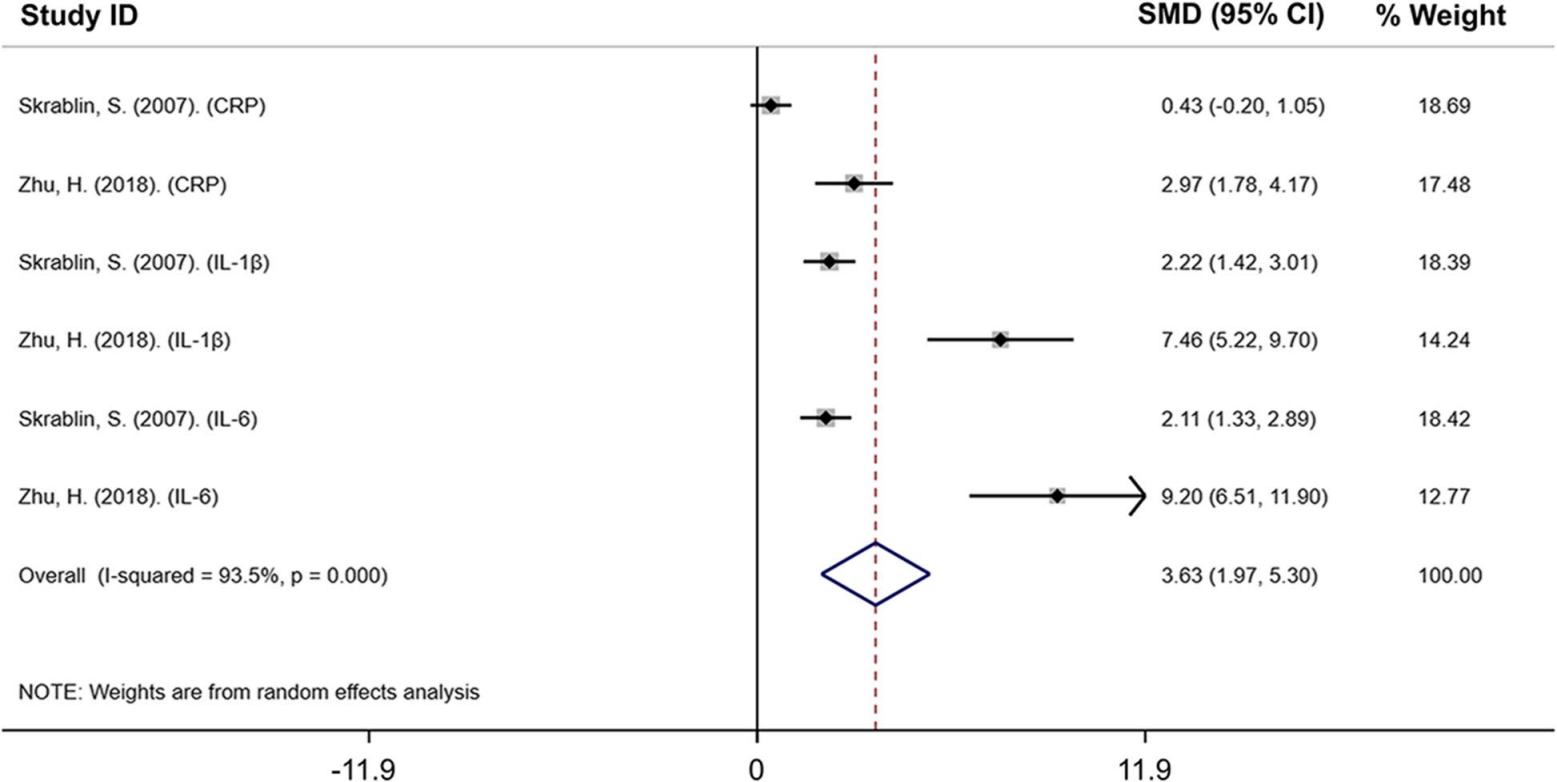
The forest plot of comparison of maternal CRP/IL-1β/IL-6 cluster between normal and PTB patients in gestational 27–34 weeks. SMD: 3.633; 95% CI: 1.971 to 5.296; *P* < 0.05, and I^2^ = 93.5%. (SMD indicated standard mean difference; CI indicated confidence interval)

The studies (Skrablin, S. 2007; Zhu, H. 2018) found that CRP, IL-1β and IL-6 these three inflammatory factors both upregulated in maternal blood of PTB patients in gestational 27–34 weeks comparing with normal [23, 24], and these two studies were included for further analysis. Based on this, the global consideration of multiple inflammatory factors at specific time for infectious PTB prediction is necessary. The forest plot indicated that CRP, IL-1β, and IL-6 all significantly higher in the maternal blood of PTB patients compared with normal (SMD: 3.633; 95% CI: 1.971 to 5.296; *P* < 0.05, and I^2^ = 93.5%) in gestational 27–34 weeks (Fig. 4). The funnel plot with pseudo 95% confidence limits was shown in Supplementary data 2A, and the sensitivity analysis shown in Supplementary data 2B did not significantly affect the overall results, supporting the function of CRP, IL-1β and IL-6 together as a reliable cluster for infectious PTB in gestational 27–34 weeks.

#### TNF/NGF family is potential cluster for predicting the occurrence of infectious PTB in gestational 25–33 weeks

**Fig. 5:**
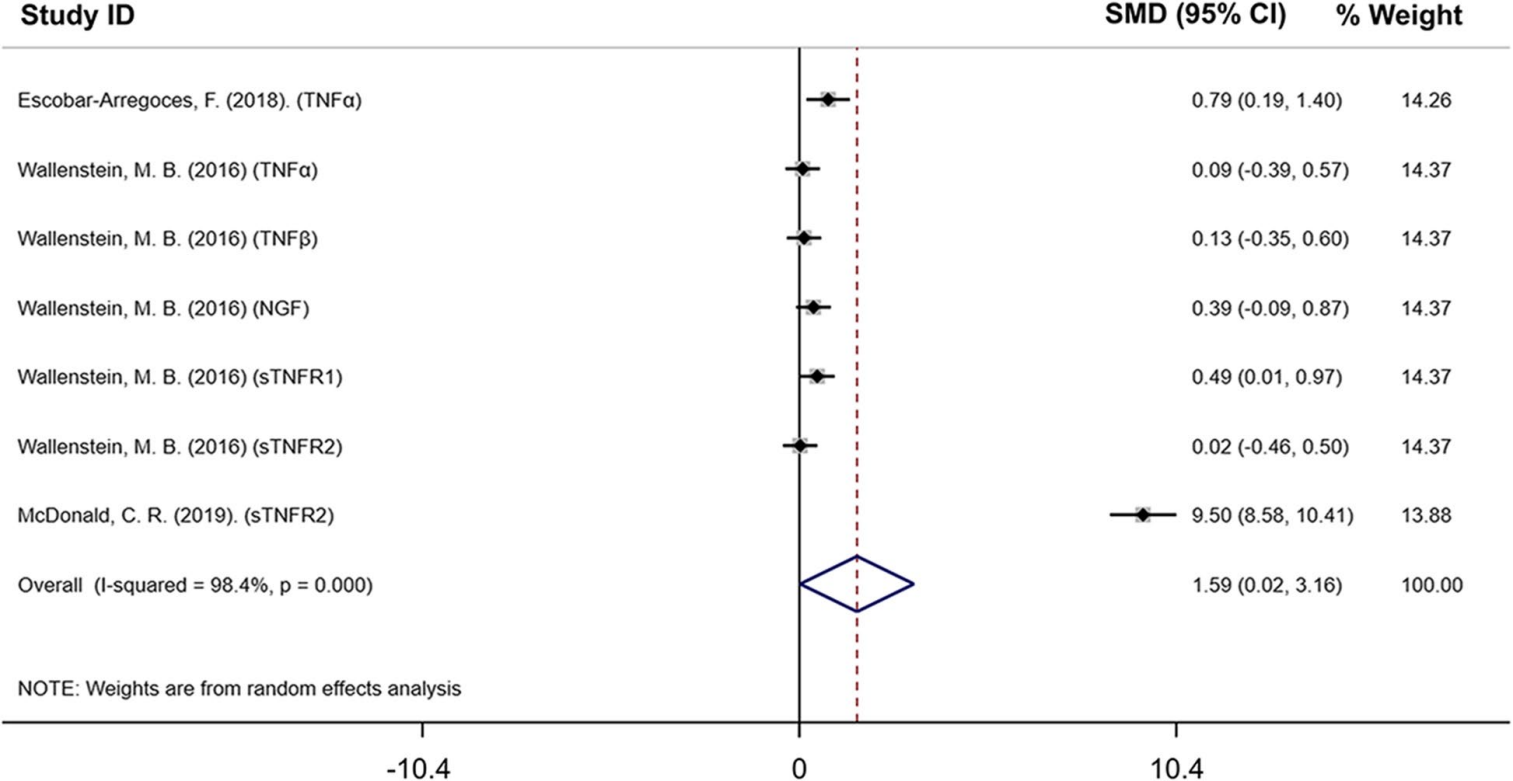
The forest plot of comparison of maternal TNF/NGF family related factors between normal and PTB patients in 25–33 weeks. SMD: 1.592; 95% CI: 0.020 to 3.163; *P* < 0.05, and I^2^ = 98.4%. (SMD indicated standard mean difference; CI indicated confidence interval)

Signals emanating from receptors of the TNF/NGF family take part in immune defense [25]. TNF, NGF, TNF receptor, and NGF receptor all belong to the TNF/NGF family. Among 17 selected references in this study, 3 references (Escobar-Arregoces, F. 2018; Wallenstein, M.B. 2016; McDonald, C. R. 2019) that related to the TNF/NGF family and analyzed maternal blood in gestational 25–33 weeks of PTB were chosen for further analysis [21, 26, 27]. Based on these, we looked at TNFα, TNFβ, NGF, sTNFR1, and sTNFR2 in these 3 studies to see if the TNF/NGF family participated in the occurrence of infectious PTB. The forest plot result showed that the TNF/NGF family participated in infectious PTB occurrence (SMD: 1.592; 95% CI: 0.020 to 3.163; *P* < 0.05, and I^2^ = 98.4%) (Figure 5). The funnel plot with pseudo 95% confidence limits was shown in Supplementary data 3A. To show the impact of each study, we did the sensitivity analysis in supplementary data 3B. The results of sensitivity analysis showed that the three studies all contributed importantly, and excluding either study would affect the statistical difference.

## Discussion

As it became attractive that inflammatory factors can be a significant trigger for labor in pregnancy [28], the number of studies examining the interaction between inflammatory factors and infectious PTB increased dramatically. Thus, a systematic study to find the maternal inflammatory biomarkers for infectious PTB is necessary.

Actually, most of the PTB related studies are retrospective, and the blood sampling time during gestation is not continuous. Clinically, maternal blood can be obtained at around 15–20 gestational weeks for Down screening, or at PTB delivery time at around 27–37 gestational weeks. If the patient shows signs of parturition very early and undergoes hospitalization, maternal blood can also be taken before 27 weeks of gestation. These are the reasons for sampling at different gestational age of infectious PTB patients among different studies.

**Fig. 6:**
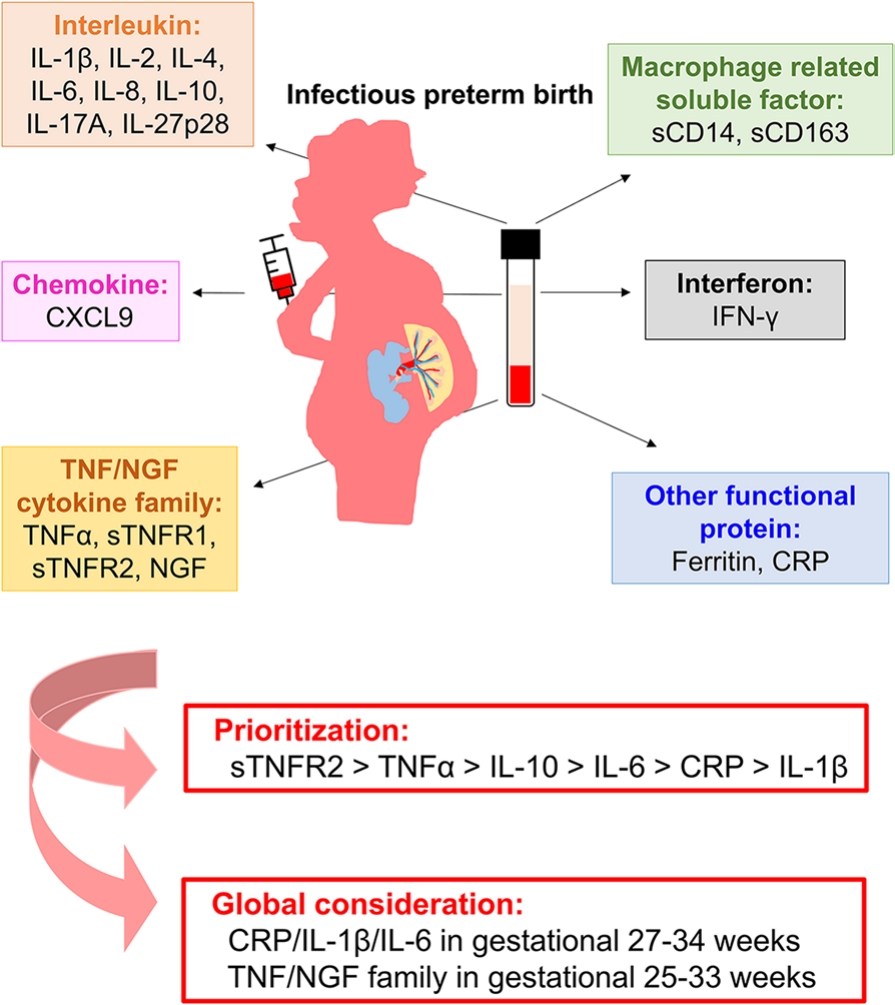
A scheme of the potential inflammatory biomarkers of infectious PTB based on the results revealed in this study. Infectious PTB was an inflammation-related disease with maternal high levels of several inflammatory factors. The prioritization of the inflammatory factors in infectious PTB prediction is sTNFR2 > TNFα > IL-10 > IL-6 > CRP > IL-1β. CRP/IL-1β/IL-6 in gestational 27–34 weeks, and TNF/NGF family in gestational 25–33 weeks, were potential biomarker clusters that specific for infectious PTB prediction. (IL-1β indicated interleukin-1 beta, IL-2 indicated interleukin-2, IL-4 indicated interleukin-4, IL-6 indicated interleukin-6, IL-8 indicated interleukin-8, IL-10 indicated interleukin-10, IL-17A indicated interleukin-17A, IL-27p28 indicated interleukin-27p28, CRP indicated C-reactive protein, TNFα indicated tumor necrosis factor α, sTNFR indicated soluble tumor necrosis factor receptor, NGF indicated nerve growth factor, CXCL9 indicated C-X-C motif chemokine ligand 9, IFN-γ indicated interferon gamma, sCD14 indicated soluble CD14 molecule, and sCD163 indicated soluble CD163 molecule)

Based on an analysis of pooled data from the included 17 references, the overall findings from this review showed that infectious PTB was an inflammation-related disease with maternal high levels of inflammatory factors. In particularly, the prioritization of the inflammatory factors in PTB prediction is sTNFR2 > TNFα > IL-10 > IL-6 > CRP > IL-1β. TNFα is a primary mediator of systemic responses to infection. The soluble receptor forms of TNFα are sTNFR1 and sTNFR2. TNFα and the soluble receptor forms increase in inflammation both with and without infection [29–31]. IL-6 has opposite effect under different inflammation environment. During the chronic inflammation and autoimmunity situation, IL-6 activity can be commonly found dysregulated. However, when inflammation occurs with infection, IL-6 increases to activate innate and adaptative immune responses [32]. From our study, CRP/ IL-1β/ IL-6 during 27–34 weeks and TNF/NGF family during 25–33 weeks can be potential biomarkers for predicting the occurrence of infectious PTB at accurate gestational age (Fig. 6).

During normal pregnancy, the healthy growth of the semi-allogeneic fetus requires the adaptive regulation of the maternal immune system to establish an immune-tolerant environment [33]. In addition, disruption of immune regulation at the maternal–fetal interface is tightly associated with various pregnancy-related diseases [34]. Decidual natural killer cells and macrophages have a key role in immune regulation at the maternal–fetal interface with the function of secreting interleukins [35–37]. Abnormal levels of IL-6, which is secreted by decidual natural killer cells [38], and IL-1β, which is secreted by macrophages [39], were found in pregnancy disorders. In addition, elevated CRP levels are also found with chronic placental inflammation. In this way, the abnormal fluctuations of the inflammatory factors, such as CRP, IL-1β, and IL-6, can reflect pregnancy disorders to some extent.

CRP, IL-1β, and IL-6 are also significantly increased in preeclamptic patients [40], so the inflammatory biomarkers for distinct different pregnancy-related diseases, such as PTB and preeclampsia, need further study. In preeclampsia, a systematic study found that CRP significantly upregulated in maternal blood in third trimester, IL-1β could be potential biomarkers in first trimester, and IL-6 upregulated in second and third trimesters [40]. In our study, we systematically found that CRP, IL-1β, and IL-6 all significantly upregulated in gestational 27–34 weeks in maternal blood of infectious PTB patients. Based on these, the expression patterns of CRP, IL-1β, and IL-6 between infectious PTB and preeclamptic maternal blood are different. CRP/IL-1β/IL-6 can be reliable cluster for infectious PTB prediction during gestational 27–34 weeks. This cluster can be special biomarker for infectious PTB, and helps to distinguished from preeclampsia.

The TNF/NGF family was first pointed out to be good candidates for infectious PTB prediction in our study, and our result indicated that the global consideration of multiple inflammatory factors could be a new direction for infectious PTB biomarkers in future studies. In this way, taking more than one biomarker into consideration for different prenatal disorders is necessary.

### Limitations

There was inevitable heterogeneity in the studies that we chose to analyze. The study population, age, parity, gestational age, and method of statistics have variations among different studies. Therefore, it is important to interpret the study results with caution. In addition, it will be better to find the biomarkers as early as possible to predict infectious PTB. However, few studies analyzed the inflammatory factors in maternal blood in the first trimester. In this way, the inflammatory biomarkers that were found in this study are much more reliable after the first trimester of pregnancy.

## Conclusion

Our study made a network meta-analysis by using the principle of the PICOS framework according to the recommendations of PRISMA to guarantee objectivity. We confirmed that infectious PTB was an inflammation-related disease with maternal high levels of inflammatory factors. In particularly, the prioritization of the inflammatory factors in infectious PTB prediction is sTNFR2 > TNFα > IL-10 > IL-6 > CRP > IL-1β. Furthermore, our innovative findings indicated that global consideration of multiple inflammatory factors, such as CRP/IL-1β/IL-6 biomarker cluster in gestational 27–34 weeks, and the TNF/NGF family in gestational 25–33 weeks, were potential biomarker clusters that specific for infectious PTB prediction. This study systematically provided evidence to both rank the inflammatory biomarkers and find several maternal inflammatory clusters at accurate gestational week for infectious PTB prediction. The global consideration of multiple inflammatory factors at accurate gestational age is highlighted.

## Supplementary information

**Additional file 1: Supplementary data 1.** Research terms of this study. **Supplementary data 2.** The funnel plot and sensitivity analysis of maternal CRP, IL-1β and IL-6 biomarker clusters between normal and PTB. **Supplementary data 3.** The funnel plot and sensitivity analysis of maternal TNF/NGF family related molecules between normal and PTB.

## Data Availability

The data of this study are available from the corresponding author upon reasonable request.

## Abbreviations

PTB: Preterm birth
IL: Interleukin
CRP: C-reactive protein
TNF: Tumor necrosis factor
sTNFR: Soluble tumor necrosis factor receptor
NGF: Nerve growth factor
IFN: Interferon
I-FABP: Intestinal fatty acid binding protein
CXCL9: C-X-C motif chemokine ligand 9
IFN-γ: Interferon gamma
sCD14: Indicated soluble CD14 molecule
sCD163: Soluble CD163 molecule
SUCRA: Surface under the cumulative ranking curve
PICOS: Intervention, comparison, outcome, and study design
PRISMA: Preferred reporting items for systematic reviews and meta-analysis
SMD: Standard mean difference
CI: Confidence interval
