# Supplementary material for: Finding the priority and cluster of inflammatory biomarkers for infectious preterm birth: a systematic review
Source: J Inflamm (Lond). 2023 Jul 24;20:25. doi: 10.1186/s12950-023-00351-0 (PMC10367376; doi:10.1186/s12950-023-00351-0)

**Supplementary data 1.** Research terms of this study

(((((((((("Premature Birth"[Mesh]) OR (Birth, Premature[Title/Abstract])) OR (Births, Premature[Title/Abstract])) OR (Premature Births[Title/Abstract])) OR (Preterm Birth[Title/Abstract])) OR (Birth, Preterm[Title/Abstract])) OR (Births, Preterm[Title/Abstract])) OR (Preterm Births[Title/Abstract])) OR (Preterm labor[Title/Abstract])) OR (Premature labor[Title/Abstract])) AND ((((("Inflammation"[Mesh]) OR (Inflammations[Title/Abstract])) OR (Innate Inflammatory Response[Title/Abstract])) OR (Inflammatory Response, Innate[Title/Abstract])) OR (Innate Inflammatory Responses[Title/Abstract]))

**Supplementary data 2.** The funnel plot and sensitivity analysis of maternal CRP, IL-1β and IL-6 biomarker clusters between normal and PTB. (A) The funnel plot with pseudo 95% confidence limits showed the standard mean difference of each study. (B) The sensitivity analysis showed the lower CI limit and upper CI limit of each study. CI indicated confidence interval.


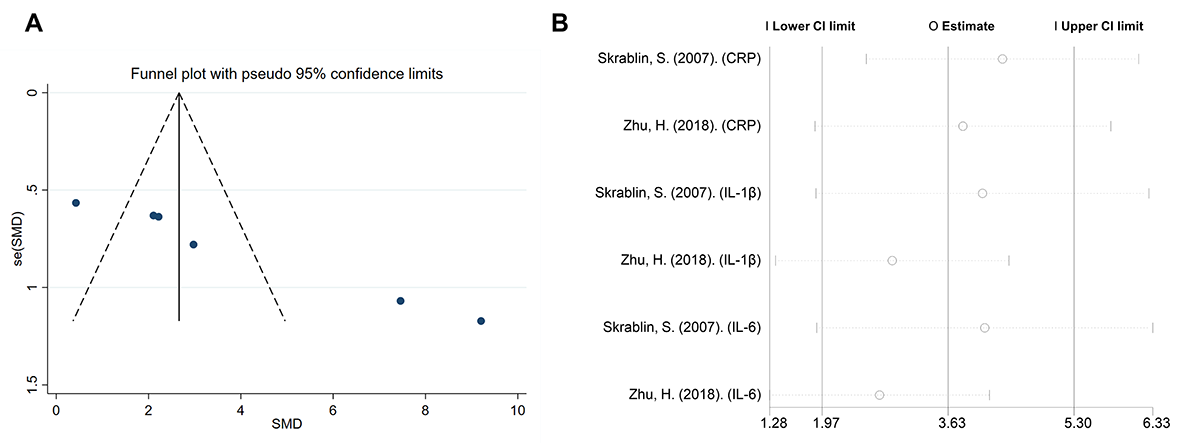


**Supplementary data 3.** The funnel plot and sensitivity analysis of maternal TNF/NGF family related molecules between normal and PTB. (A) The funnel plot with pseudo 95% confidence limits showed the standard mean difference of each study. (B) The sensitivity analysis showed the lower CI limit and upper CI limit of each study. CI indicated confidence interval.


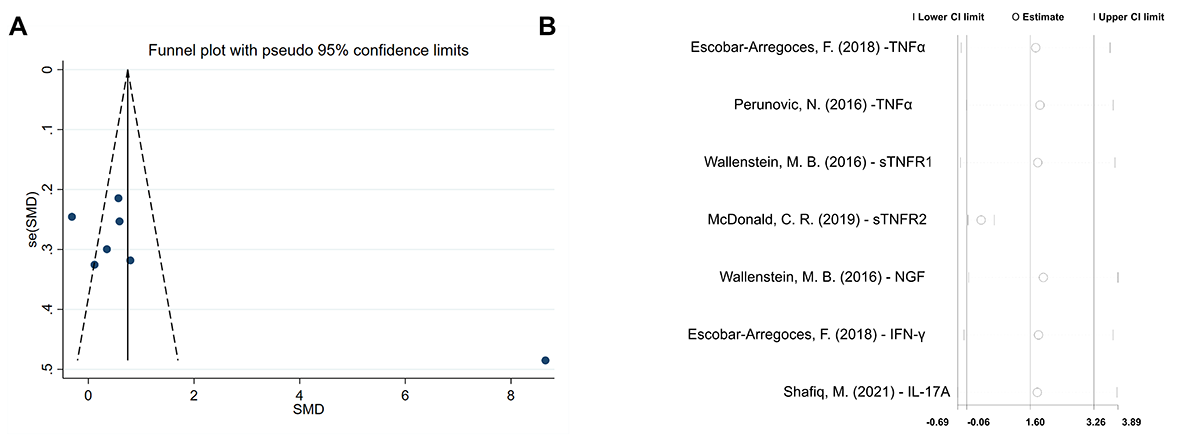

Supplement: Supplementary file 1 — Additional file 1: Supplementary data 1. Research terms of this study. Supplementary data 2. The funnel plot and sensitivity analysis of maternal CRP, IL-1β and IL-6 biomarker clusters between normal and PTB. Supplementary data 3. The funnel plot and sensitivity analysis of maternal TNF/NGF family related molecules between normal and PTB. [file 12950_2023_351_MOESM1_ESM.docx]
